# Zinc Finger-Homeodomain Transcriptional Factors (ZF-HDs) in Wheat (*Triticum aestivum* L.): Identification, Evolution, Expression Analysis and Response to Abiotic Stresses

**DOI:** 10.3390/plants10030593

**Published:** 2021-03-22

**Authors:** Hao Liu, Ying Yang, Linsheng Zhang

**Affiliations:** 1 College of Life Science, Northwest A&F University, Yangling 712100, China; liuhao_1990@126.com; 2 College of Agriculture, Ludong University, Yantai 264011, China; 3 College of Nursing, Weinan Vocational & Technical College, Weinan 714000, China; yy820923@sina.com

**Keywords:** *ZF-HD* genes, genome-wide identification, gene expression, wheat (*Triticum aestivum* L.)

## Abstract

Zinc finger-homeodomain transcriptional factors (ZF-HDs), a kind of plant-specific transcription factor, play important roles in plant growth, development and various stress responses. In this study, the genome-wide analysis of the *ZF-HD* gene family was performed in wheat. A total of 37 *TaZF-HD* genes were identified in *T. aestivum* and classified into six groups. The results of a synteny analysis showed that gene replication events contributed to the expansion of the *TaZF-HD* gene family. The *TaZF-HD* paralogous gene pairs with similar chromosomal locations in different subgenomes had similar expression patterns. *TaZF-HD*s were highly induced under PEG (polyethylene glycol), NaCl and cold stress but not induced under heat stress. Gene ontology (GO) annotation and protein-protein interactions suggested that TaZF-HD proteins may participate in various biological processes of plants. These results increase our understanding of *ZF-HD* genes and provide robust candidate genes for future functional investigations aimed at crop improvement.

## 1. Introduction

Transcription factors (TFs) have been confirmed to play central roles in the regulatory networks of plant growth, development and stress responses through binding to specific *cis*-elements [1,2]. For example, WRKY, NAC and GRF transcription factors are associated with seed development, leaf senescence, plant proliferation and expansion [3,4,5] whereas MYB, WRKY and NAC transcription factors can participate in the response of plants to biotic and abiotic stresses [6,7,8]. Zinc finger-homeodomain (ZF-HD) proteins are a kind of plant-specific transcription factor that plays vital roles in plant growth, development and various stress responses [9,10].

ZF-HD proteins contain a homeodomain (HD) domain and a C2H2-type zinc finger (ZF) domain [11,12]. The HD domain is a well-characterized DNA binding domain (BD), which can be involved in the growth and development of plants by binding to DNA to regulate the expression levels of target genes [11,13]. Most homeodomain proteins have other domains that participate in protein-protein interaction and other functions [14]. The ZF domain widely exists in regulatory proteins and participates in DNA binding and protein-protein interactions [15]. ZF-HD proteins usually bind to specific DNA sequences with a core consensus of ATTA and form homodimers and heterodimers [9].

ZF-HD proteins were first identified in the C4 plant *Flaveria* as a potential regulator of the gene encoding C4 phosphoenolpyruvate carboxylase (PEPCase) [13]. Subsequently, *ZF-HD* genes were identified in several plant species such as *Arabidopsis thaliana* [11], tomato (*Solanum lycopersicum*) [16], cotton (*Gossypium hirsutum*) [17], Chinese cabbage [12] and Tartary buckwheat (*Fagopyrum tataricum*) [18]. In *Arabidopsis*, ZFHD10 recruits TANDEM ZINC-FINGER PLUS3 (TZP) to bind to light-regulated elements and regulate hypocotyl elongation [19]. *ZHD1* is induced after drought, salinity and abscisic acid (ABA) treatments and specifically binds to the promoter region of EARLY RESPONSE TO DEHYDRATION STRESS1 (*ERD1*) [10]. In addition, the overexpression of *NAC* and *ZHD1* enhances drought tolerance in *Arabidopsis* [10]. In soybeans, the expression of *GmZF-HD1* and *GmZF-HD2* are induced after pathogen inoculation and bind to the promoter region of the calmodulin subtype 4 gene (*GmCaM4*) [20]. Interestingly, *mini zinc finger* (*MIF*) genes identified in the *ZF-HD* gene family of *Arabidopsis* encode proteins with the ZF domain of ZF-HD proteins but without the HD domain [11]. Phylogenetic and sequence analyses of *ZF-HD* genes demonstrated that both *ZFD*s and *MIF*s are land plant-specific and they belong to two different groups of the *ZF-HD* gene family [11]. However, the origin and evolution of the *ZHD* and *MIF* genes remain unclear. *MIF*s might be derived from *ZHD*s by losing the HD domain; alternatively, *ZHD*s might have originated from *MIF*s by gaining the HD domain [11].

*ZF-HD* genes have been identified in several plant species; however, a genome-wide identification of *ZF-HD* genes in wheat has not been performed. Fortunately, whole genome sequence data of the bread wheat “Chinese Spring” were published [21], making it possible to identify and analyze the *ZF-HD* gene family in wheat. Therefore, in this study, we performed a genome-wide analysis of *ZF-HD* genes in wheat to characterize their sequences, evolutionary relationships and expression patterns in different tissues and under various abiotic stress treatments.

## 2. Results

### 2.1. Characteristics and Phylogenetic Analysis of TaZF-HDs in T. aestivum

**Figure 1:**
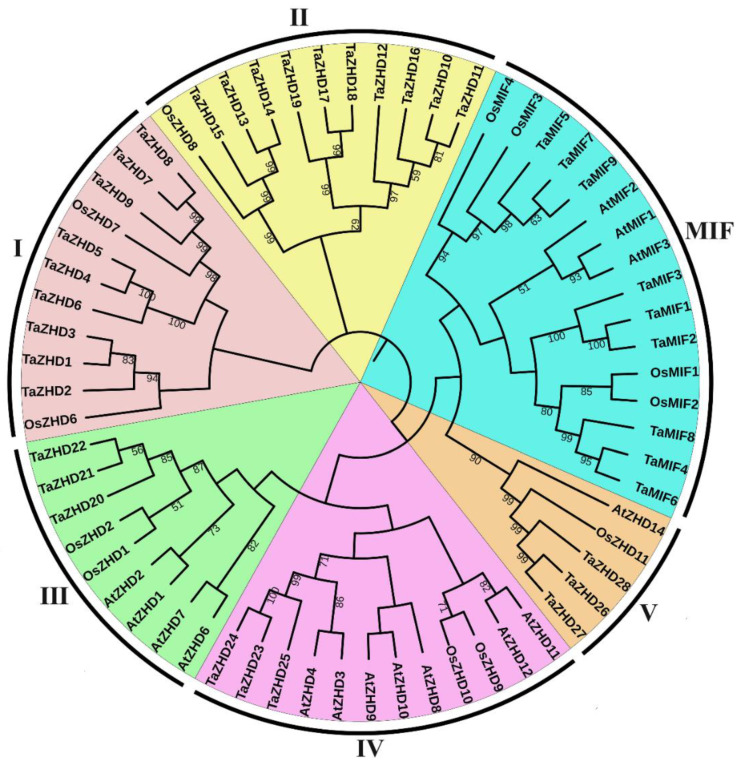
The neighbor-joining (NJ) phylogenetic tree of ZF-HD proteins. The tree was constructed with amino acid sequences of identified ZF-HD proteins from in *A. thaliana* (At), *O. sativa* (Os) and *T. aestivum* (Ta) and bootstrap values of 1000 replicates. Different groups of ZF-HD proteins are distinguished by different colors.

**Figure 2:**
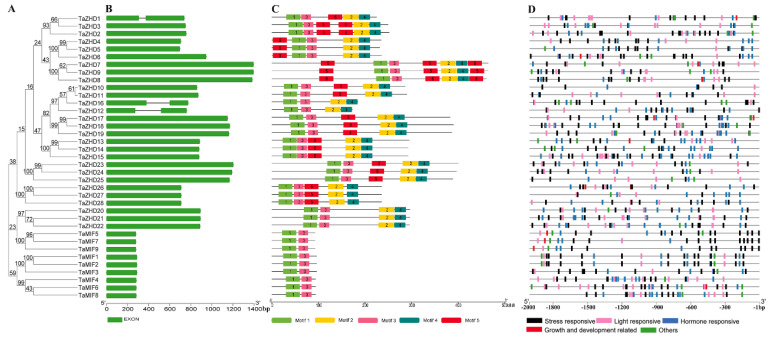
Phylogenetic relationships (**A**), exon-intron structures (**B**), conserved domains (**C**) and *cis*-elements analysis of *TaZF-HD* genes. (**B**) Green boxes and black lines indicated exons and introns, respectively. (**C**) Conserved domain compositions of TaZF-HD proteins in wheat. Motif 1 to motif 5 are shown in the different colors. (**D**) Hormone-responsive elements, stress-responsive elements, growth and development related elements, light-responsive elements and other elements with unknown functions are shown by different colors.

A total of 37 ZF-HD proteins were identified from a wheat genome based on a Hidden Markov Model (HMM) search of a ZF-HD dimerization region (PF04770) (Table S1). Based on chromosome locations and the phylogenetic relationship of *TaZF-HD*s, we renamed them from *TaZHD1* to *TaZHD28* and *TaMIF1* to *TaMIF9*. The 37 predicted *TaZF-HD* genes encoded polypeptides of 93–465 amino acids in length with predicted molecular weights ranging from 9.84 (TaMIF6) to 50.6 (TaZHD9) kDa and the isoelectric points (pI) ranged from 6.17 (TaMIF1) to 9.89 (TaZHD3). The calculated grand average of hydropathy index (GRAVY) values of all TaZF-HDs were below zero (−0.309 to −1.14), suggesting that they were hydrophilic. A subcellular localization prediction indicated that TaZF-HD proteins were all located in the nuclear. The phylogenetic tree was constructed with TaZF-HD, AtZF-HD and OsZF-HD proteins (Table S2). The results indicated that TaZF-HD proteins were divided into six groups (Figure 1 and Figure 2A), which were consistent with previous phylogenetic analyses of plant ZF-HD proteins [16,18]. Group II and MIF were the largest with 10 and 9 members, respectively. Group I, III, IV and V included 7, 3, 3 and 3 members, respectively (Figure 1 and Table S1).

### 2.2. Gene Structures, Conserved Motifs and Cis-Elements Analysis of TaZF-HDs

**Figure 3:**
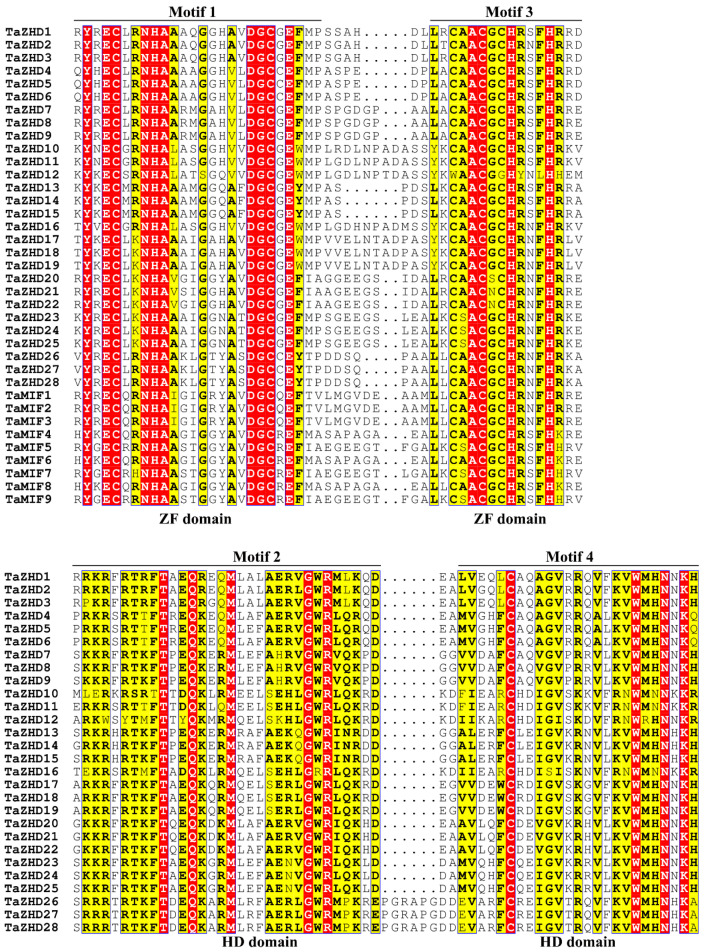
Multiple sequence alignment of the conserved domains of the members of the *TaZF-HD* gene family in wheat. Motifs 1 and 3 were ZF domains and motifs 2 and 4 were HD domains.

To investigate the structural characteristics of the *TaZF-HD* genes, the exon-intron structures (Figure 2B) and conserved motifs (Figure 2C) of 37 *TaZF-HD* genes were analyzed. The exon-intron structures analysis indicated that most of the *TaZF-HD* genes did not include introns and only three *TaZF-HD*s (*TaZHD1*, *12* and *16*) had one intron (Figure 2B and Table S1). To further investigate the diversity of TaZF-HD genes, the conserved motifs were analyzed using the MEME (Multiple Em for Motif Elicitation) online server (Figure 2C). Finally, five motifs were identified among of 37 TaZF-HD genes and named motif 1 to motif 5 (Figure 2C and Figure S1). All of the TaZF-HD proteins contained motif 1 and 3, which were typical ZF domains. Motif 2 and 4, the typical HD domains, were present in all TaZHDs (TaZHD1–TaZHD28) but were absent in TaMIFs (TaMIF1–TaMIF9) (Figure 2C and Figure 3). These results verified the reliability of the identified members of the *TaZF-HD* gene family. The same group of *TaZF-HD*s had similar exon-intron structures and conserved motifs and *TaZF-HD*s with closer evolutionary relationships had more similar numbers and lengths of exons and conserved motifs (Figure 2).

The presence of multiple different *cis*-elements in the gene promoters might indicate that these genes perform different functions. To explore the *cis*-elements in the promoters of *TaZF-HD* genes, 2 kb of the upstream genomic sequence of each gene transcription start site (TSS) were extracted and then searched on the PlantCARE database to identify and count the *cis*-elements. These *cis*-elements could be divided into five categories: (i) hormone-responsive elements such as ABRE (ABA-responsive element), TGA-element (auxin-responsive element), CGTCA-motif (methyl jasmonate-responsive element), TGACG-motif (methyl jasmonate-responsive element), P-box (gibberellin-responsive element), TCA-element (salicylic acid-responsive element); (ii) stress-responsive elements such as DRE (dehydration-responsive element), LTRE (low temperature-responsive element), MBS (MYB binding site), MYC, STRE (stress-responsive element), W-box (WRKY transcription factor binding site), WRE3 (wounding-responsive element); (iii) growth and development related elements, CAT-box (*cis*-element related to meristem expression), circadian (*cis*-element related to circadian control) and RY-elements (*cis*-element related to seed-specific regulation); (iv) light-responsive elements such as ACE (AC-rich element), GA-motif, G-box, I-box; (v) other elements with unknown functions (Figure 2D). *TaZF-HD*s with closer evolutionary relationships showed a similar distribution of *cis*-elements in their promoters such as *TaZHD20-TaZHD21* and *TaMIF1*-*TaMIF2*. These results indicated that the *TaZF-HD* genes may participate in the growth and development and respond to various stresses in wheat.

### 2.3. Chromosomal Location, Synteny and Ka/Ks Analysis of TaZF-HDs

**Figure 4:**
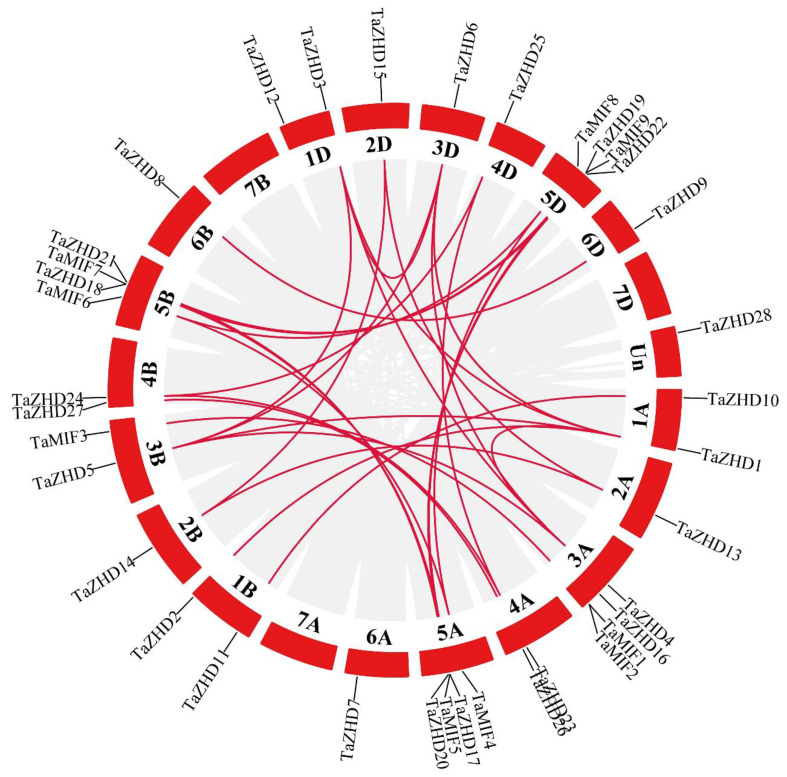
Chromosomal localizations and syntenic relationships among *TaZF-HD* genes in *T. aestivum*. Gray lines in the background indicate the collinear blocks among *T. aestivum* and red lines highlight the syntenic *TaZF-HD* gene pairs.

The chromosomal locations of the identified *TaZF-HD* genes in wheat were mapped to the corresponding chromosomes by the MapChart and circos tools (Figure 4 and Figure S2). *TaZF-HD* genes were distributed in chromosome 1, 2, 3, 4, 5, 6 and Un. Chromosome 5, which was the chromosome with the most *TaZF-HD* genes, contained twelve *TaZF-HD* genes and chromosome 7 had no *TaZF-HD* genes. *TaZF-HD* genes were approximately evenly and similarly distributed in the A, B and D subgenomes.

Synteny analysis suggested that 36 paralogous gene pairs were found among 37 *TaZF-HD* genes in wheat with the most gene duplication events on chromosome 1A and only one on chromosome 6B and 6D, respectively (Figure 4 and Table S3). Moreover, 32 *TaZF-HD*s had undergone WGD (whole genome duplications) or segmental duplication events whereas only one gene (*TaMIF2*) was a tandem replication. These results showed that most *TaZF-HD* genes might be produced by fragment replication events and these replication events played a vital function in the evolution of *TaZF-HD* genes. To investigate the selective pressure on the duplicated *TaZF-HD* genes, the non-synonymous (Ka) and synonymous substitution (Ks) values were calculated for the 36 paralogous gene pairs (Table S3). The value of Ka/Ks = 1 meant that genes experienced a neutral selection, Ka/Ks > 1 indicated a positive selection and Ka/Ks < 1 suggested a purifying/negative selection [22]. The values of Ka/Ks from all 36 paralogous gene pairs were less than 1, which indicated that these *TaZF-HD* genes had undergone a strong purifying/negative selection pressure with little changed after duplication.

**Figure 5:**
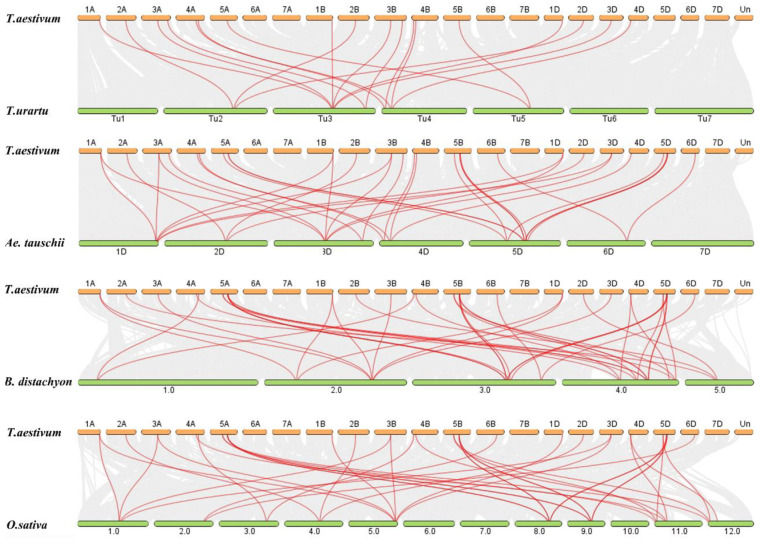
Syntenic relationships between *TaZF-HD* genes in *T. aestivum* with other *ZF-HD* genes in four representative plant species. Gray lines in the background indicate the collinear blocks within *T. aestivum* and other plant genomes and red lines highlight the syntenic *ZF-HD* gene pairs.

To further investigate the synteny relationships of *ZH-HD* genes between the *T. aestivum* (AABBDD, hexaploid) with *T. urartu* (AA, diploid), *Ae. tauschii* (DD, diploid), *B. distachyon* (diploid) and *O. sativa* (diploid), a Multiple Collinearity Scan toolkit (MCScanX) was used to identify the orthologous genes among these released plant genomes (Figure 5 and Table S4). We identified 18, 35, 41 and 39 orthologous gene pairs between *TaZF-HD*s with other *ZH-HD* genes in *T. urartu*, *Ae. tauschii*, *B. distachyon* and *O. sativa*, respectively. The results showed that 18, 29, 28 and 25 *TaZF-HD*s genes were collinear with *ZH-HD* genes in *T. urartu*, *Ae. tauschii*, *B. distachyon* and *O. sativa*, respectively. A few *TaZF-HD* genes had at least two pairs of orthologous genes such as *TaZHD1*, *TaZHD2*, *TaZHD5* and *TaMIF8*, which might have played a vital function in the evolution of *ZF-HD* genes. These results indicated that *TaZH-HD* genes in wheat might be derived from orthologous genes in other plant species.

### 2.4. Expression Patterns of TaZF-HD Genes in Different Tissues

**Figure 6:**
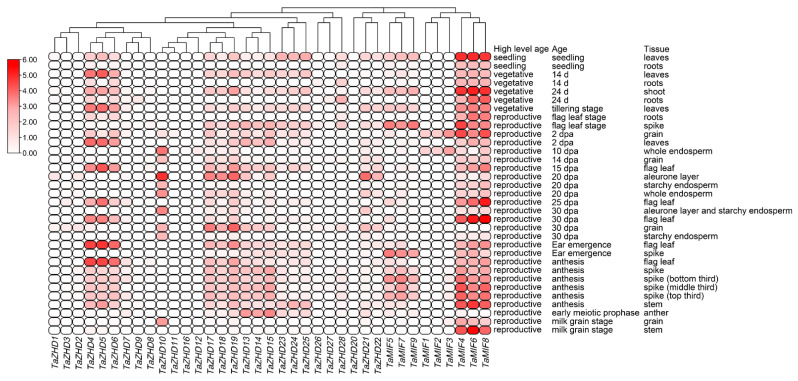
Tissue-specific expression patterns of the *TaZF-HD* genes in various wheat tissues. The log_2_ of TPM (transcripts per million) values were calculated by RNA-seq data to show the expression levels of the *TaZF-HD* genes in wheat.

To investigate the tissue-specific expression patterns of the *TaZF-HD* genes in wheat, the RNA-seq data in wheat seedling, vegetative and reproductive stages were obtained from the expVIP website (Figure 6 and Table S5). Most *TaZF-HD* genes exhibited tissue-specific expression patterns and could be detected in the leaves, spikes and grains but not expressed in the roots. Many paralogous gene pairs with similar chromosomal locations in different subgenomes had more similar expression patterns; for example, *TaZHD1*, *TaZHD2* and *TaZHD3* were mainly expressed in grains at 30 days post-anthesis (dpa); *TaMIF1*, *TaMIF2* and *TaMIF3* were highly expressed in grains at 2 dpa and in a whole endosperm at 10 dpa. Fewer *TaZF-HD* gene pairs were expressed in most tissues; for example, *TaMIF4*, *TaMIF6* and *TaMIF8* exhibited higher expression levels in almost all tissues except in grains at 30 days post-anthesis (dpa); *TaZHD4*, *TaZHD5* and *TaZHD6* were also highly expressed in most tissues. However, a few paralogous gene pairs had different expression patterns in various tissues, e.g., *TaZHD10* had higher expression levels in the whole endosperm, aleurone layer and starchy endosperm and *TaZHD11* was almost not expressed (Figure 4 and Figure 6).

**Figure 7:**
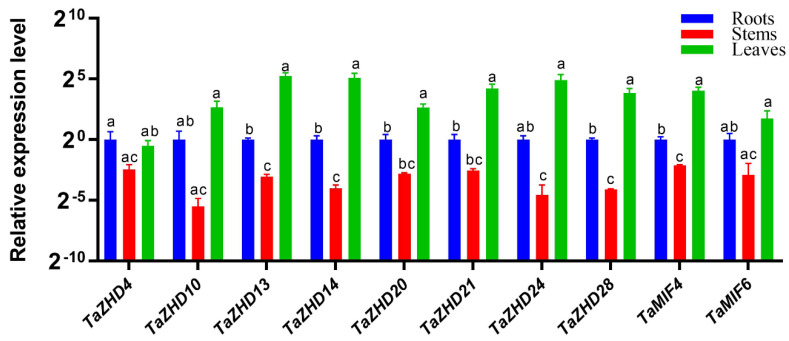
Expression levels of *TaZF-HD* genes in the roots, stems and leaves of wheat seedlings. The expression level of the wheat *actin* gene was used as the internal control to standardize the RNA samples for each reaction. The values are the mean ± SE from three samples. Statistical significance was determined by a student’s *t*-test and different lowercase letters indicate significant differences (*p* < 0.05) for each parameter.

We also determined the expression profiles of ten *TaZF-HD* genes belonging to I (*TaZHD4*), II (*TaZHD10*, *TaZHD13* and *TaZHD14*), III (*TaZHD20* and *TaZHD21*), IV (*TaZHD24*), V (*TaZHD28*) and MIF (*TaMIF4* and *TaMIF6*) groups in the roots, stems and leaves of wheat seedlings by real-time PCR (Figure 7). The results indicated that nine of these ten *TaZF-HD* genes (*TaZHD10*, *TaZHD13*, *TaZHD14*, *TaZHD20*, *TaZHD21*, *TaZHD24*, *TaZHD28*, *TaMIF4* and *TaMIF6*) had the highest expression levels in the leaves followed by the roots. The expression level of *TaZHD4* was the highest in the roots followed by the leaves. All of these ten *TaZF-HD* genes had the lowest expression levels in the stems. These results suggested that these ten *TaZF-HD* might play important roles in the development process of the leaves.

### 2.5. Expression Patterns of TaZF-HD Genes under Abiotic Stresses

**Figure 8:**
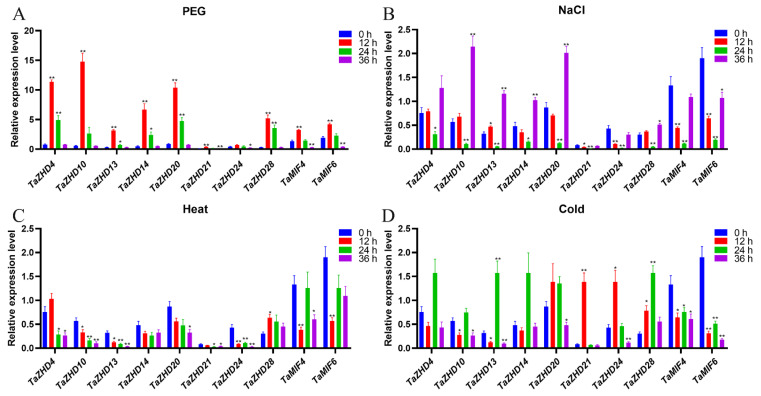
Expression patterns of *TaZF-HD* genes in response to PEG (polyethylene glycol) (**A**), NaCl (**B**), heat (**C**) and cold (**D**) treatments determined by real-time PCR. The expression level of the wheat *actin* gene was used as the internal control to standardize the RNA samples for each reaction. The values are the mean ± SE from three samples and significant differences were indicated as (*) *p* < 0.05 and (**) *p* < 0.01.

To further investigate the function of *TaZF-HD* genes, real-time PCR was used to detect the expression profiles of ten *TaZF-HD* genes (*TaZHD4*, *TaZHD10*, *TaZHD13*, *TaZHD14*, *TaZHD20*, *TaZHD21*, *TaZHD24*, *TaZHD28*, *TaMIF4* and *TaMIF6*) under PEG (polyethylene glycol), NaCl and heat and cold stresses in wheat leaves at the seedling stage (Figure 8). In PEG treatment conditions, all *TaZF-HD* genes were initially up-regulated and then down-regulated and reached the highest expression level at 12 h (2.2- to 25.9-fold vs. the control). Under NaCl stress, the expression of six *TaZF-HD* genes (*TaZHD4*, *TaZHD10*, *TaZHD13*, *TaZHD14*, *TaZHD20* and *TaZHD28*) peaked at 36 h after treatment (1.7- to 3.8-fold vs. the control). *TaZHD21*, *TaZHD24*, *TaMIF4* and *TaMIF6* were down-regulated after salt stress treatment compared with the control. Under heat stress, *TaZHD4* and *TaZHD28* were up-regulated and other *TaZF-HD* genes were down-regulated compared with the control. Under cold stress, eight *TaZF-HD* genes (*TaZHD4*, *TaZHD10*, *TaZHD13*, *TaZHD14*, *TaZHD20*, *TaZHD21*, *TaZHD24* and *TaZHD28*) were the most highly expressed at 12 h or 24 h (1.3- to 16.7-fold) compared with the control. *TaZHD4* and *TaZHD28* were up-regulated under all abiotic stresses. Paralogous genes also had similar expression patterns under abiotic stress, e.g., *TaMIF4* and *TaMIF6* had almost consistent expression patterns under PEG, NaCl, heat or cold stress.

### 2.6. GO Annotation Analysis and Protein-Protein Interactions of TaZF-HDs

**Figure 9:**
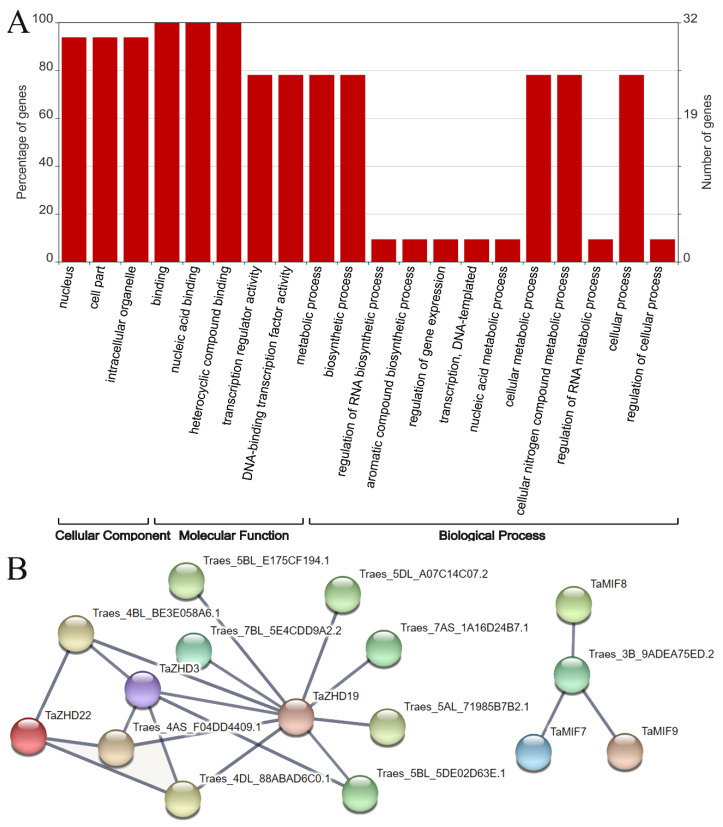
Gene ontology (GO) annotation (**A**) and protein-protein interactions (**B**) analysis of TaZF-HD proteins.

Thirty-two of the thirty-seven TaZF-HD proteins could be annotated by gene ontology (GO), which were contributed to understand the function from molecular levels. The 32 TaZF-HD proteins were assigned with 20 GO terms belonging to the cellular component, molecular function and biological process (Figure 9A). Among these 32 TaZF-HD proteins, 30 TaZF-HDs were located in the nucleus (GO:0005634). Under the molecular function category, all of the TaZF-HDs were involved in nucleic acid binding (GO:0003676) and 25 TaZF-HDs could participate in DNA binding transcription factor activity (GO:0003700). Under the biological process, 25 TaZF-HDs were involved in the cellular metabolic process (GO:0044237) and the cellular nitrogen compound metabolic process (GO:0034641).

To investigate protein-protein interactions between TaZF-HDs and other wheat proteins, a network was constructed using the STRING database (Figure 9B and Table S6). According to the predicted results, we identified six TaZH-HDs interacting with 10 other wheat proteins. TaZHD19 could interact with TaZHD3 and nine other wheat proteins, which were homeodomain-leucine zipper transcription factors (Traes_4AS_F04DD4409.1, Traes_4BL_BE3E058A6.1, Traes_4DL_88ABAD6C0.1, Traes_5BL_5DE02D63E.1), a mediator of RNA polymerase II transcription subunit 25 (Traes_5AL_71985B7B2.1, Traes_5BL_E175CF194.1, Traes_5DL_A07C14C07.2), cytochrome P450 (Traes_7AS_1A16D24B7.1) and macrophage migration inhibitory factor (Traes_7BL_5E4CDD9A2.2), suggesting that TaZHD19 played a pivotal role in the regulation of protein networks. TaZHD3 and TaZHD22 also interacted with three and four kinds of homeodomain-leucine zipper transcription factors, respectively. TaMIF7, TaMIF8 and TaMIF9 could all interact with the GATA-type zinc finger protein (Traes_3B_9ADEA75ED.2). These results provided a valuable foundation for future functional investigations of *TaZF-HD* genes.

## 3. Discussion

### 3.1. Evolution and Expansion of the TaZF-HD Gene Family in Wheat

The members of the *ZF-HD* gene family have been reported in many plants but they have not been genome-wide identified in wheat. Previous studies indicated that *ZF-HD* genes only existed in land plants and expanded during angiosperm evolution [11,12]. To further investigate the synteny relationships of *ZH-HD* genes between the wheat and other plant species, we identified 18, 35, 41 and 39 orthologous gene pairs between *TaZF-HD*s with other *ZH-HD* genes in *T. urartu*, *Ae. tauschii*, *B. distachyon* and *O. sativa*, respectively (Figure 5 and Table S4). Moreover, *T. urartu* (AA, diploid) and *Ae. tauschii* (DD, diploid) were the source of wheat (AABBDD, hexaploid) A and D subgenomes. The synteny analysis indicated that six orthologous gene pairs between *T. urartu* with a wheat A subgenome were located on the same chromosomes with one on 2A, two on 3A, two on 4A and one on 5A. Meanwhile, nine orthologous gene pairs between *Ae. tauschii* with a wheat D subgenome were located on the same chromosomes with one on 1D, one on 2D, one on 3D, one on 4D, four on 5D and one on 6D (Figure 5 and Table S4). These *TaZF-HD* genes might be derived from orthologous genes in *T. urartu* and *Ae. tauschii* with the occurrence of natural hybridization events. Furthermore, more orthologous gene pairs were identified between wheat with *B. distachyon* and *O. sativa*, which suggested that *TaZF-HD*s and other *ZH-HD* genes in *B. distachyon* and *O. sativa* might be derived from a common ancestor with a long-term evolutionary process.

The gene duplication events including tandem, segmental and whole genome duplications are primary driving forces of the expansion of the gene family in plant genome evolution [23,24]. In this study, we identified 37 *TaZF-HD* genes in wheat including 28 *TaZF-HD* genes and nine *TaMIF* genes (Figure 1 and Table S1). The number of *TaZF-HD* genes was relatively higher than that identified in *Arabidopsis* (17) [11], tomato (22) [16], Chinese cabbage (31) [12] and Tartary buckwheat (20) [18] and the same as that in cotton (37) [17], suggesting that genome duplication events might have contributed to the expansion of *TaZF-HD* genes in wheat. Thirty-six paralogous gene pairs were identified in wheat, which all had undergone WGD or segmental duplication events and a strong purifying selection pressure (Figure 4 and Table S3). In conclusion, WGD or segmental duplications played vital roles in the evolution and expansion of the *TaZF-HD* genes.

### 3.2. Expression and Function Analysis of TaZF-HD Genes in Wheat

*ZF-HD* genes participate in various biological processes and play crucial roles in plant growth, development and stress responses [9,10]. The tissue-specific expression profiles usually reveal their corresponding biological functions [25,26]. In *Arabidopsis*, most *ZF-HD* genes are expressed in floral tissues, indicating a likely regulatory role during floral development [11]. *TaZFHD1*, described as *TaZHD19* in this study, was differentially expressed during spike development with a preferential expression during “half emerged”, “completely emerged” and “half anthesis” stages [27]. In this study, *TaZHD19* also had a higher expression level in the spike during the reproductive stage (Figure 6). These results indicated that *TaZFD19* might be involved in wheat inflorescence development and/or pollination [27]. Most *TaZF-HD* genes exhibited relatively higher expression levels in the leaves, spikes and grains and lower expression levels in the roots (Figure 6 and Figure 7), which indicated *TaZF-HD*s might play an important function in their growth and development. Paralogous gene pairs with similar chromosomal locations in different subgenomes had more similar expression patterns, e.g., *TaZHD1*, *TaZHD2* and *TaZHD3*, indicating that they might have redundant functions in regulating plant growth and development (Figure 4 and Figure 6).

Previous studies have shown that *ZF-HD* genes were induced by drought and high salinity and an overexpression of *NAC* and *ZHD1* activated the expression of *ERD1* and enhanced drought tolerance in *Arabidopsis* [10]. Most *TaZF-HD* genes were also highly expressed after PEG, NaCl and cold treatments but not expressed under heat stress (Figure 8). Similarly, *VvZHD* genes were up-regulated in response to dehydration or high salinity stresses in grapes [28]. *SlZHD18* was obviously induced by drought, NaCl and cold treatments but not by heat stress [16].

According to previous studies, ZF-HD proteins can form homodimers and heterodimers or interact with other proteins to regulate plant growth, development and stress responses [9,10]. In *Arabidopsis*, ZFHD10 interacted with TZP protein to regulate hypocotyl elongation [19]. LlZHFD4 interacted with NAC transcription factor LlNAC2 to regulate the stress tolerance of the tiger lily [29]. The predicted results of the STRING database showed that TaZHD3 might interact with TaZHD19 (Figure 9B and Table S6); however, *TaZHD3* was not co-expressed with *TaZHD19* under abiotic stress (Figure S3) suggesting that *TaZHD3* and *TaZHD19* might perform other unknown functions in wheat growth and development via interacting with each other. *TaMIF7*, *8* and *9* had higher expression levels in the spike and might interact with Traes_3B_9ADEA75ED.2 (GATA-type zinc finger protein) suggesting that they might be involved in regulating wheat spike development via interacting with the GATA-type zinc finger protein (Figure 6 and Figure 8B). TaZH-HD genes also interacted with the homeodomain-leucine zipper transcription factor and cytochrome P450, etc. These results provide valuable foundations for future functional investigations of *TaZF-HD* genes and breeding new wheat varieties.

## 4. Materials and Methods

### 4.1. Identification of the ZF-HD Family Genes

The genome sequence of *T. aestivum* was downloaded from the EnsemblPlant database (http://plants.ensembl.org/index.html). The Hidden Markov Model (HMM) profiles (http://pfam.xfam.org) of the ZF-HD dimerization region (PF04770) were obtained from the Pfam database (http://pfam.xfam.org) and were used to HMM search against the local genome database of *T. aestivum* using TBtools [30]. All of the identified TaZF-HD candidates were submitted to the Pfam database (http://www.ebi.ac.uk/Tools/hmmer/) to confirm the ZF-HD protein conserved domains. We then retrieved 37 *TaZF-HD* genes. The physiological and biochemical parameters of the TaZF-HD proteins were analyzed by the ProtParam tool (http://web.expasy.org/protparam/) and the subcellular localization of the TaZF-HD proteins was predicted using the Plant-mPLoc (http://www.csbio.sjtu.edu.cn/bioinf/plant-multi/) and ProtComp 9.0 tools (http://www.softberry.com/berry.phtml?topic=protcomppl&group=programs&subgroup=proloc).

### 4.2. Phylogenetic Relationships, Gene Structures, Conserved Motifs and Cis-Elements Analysis

The phylogenetic tree was constructed by the neighbor-joining (NJ) method with 1000 bootstrap replicates in the MAFFT and ITOL online service [31,32]. The exon-intron structures were identified using the Gene Structure Display Server (GSDS) (http://gsds.cbi.pku.edu.cn/) by comparing CDS and genomic DNA sequences [33]. The conserved motifs were annotated using the MEME online server (http://meme-suite.org/index.html). The promoter sequences, which were 2000 bp upstream of the transcription start site (TSS) of the *TaZF-HD* genes, were acquired from the *T. aestivum* database and the *cis*-elements in the promoters were analyzed in the PlantCARE database [34].

### 4.3. Chromosomal Location, Synteny and Ka/Ks Analysis

The chromosomal locations of each *TaZF-HD* gene were obtained according to genome annotation data and then marked on the chromosomes using the MapChart and circos [35,36]. Multiple collinear scanning toolkits (MCScanX) were used to detect the gene replication events [37]. TBtools was used to determine the Ka and Ks of the syntenic gene pair with the Nei-Gojobori (NG) method [30].

### 4.4. Gene Ontology Annotation and Protein-Protein Interactions Analysis

GO annotation of TaZF-HD proteins was analyzed using the OmicsBox tool (https://www.biobam.com/) and displayed by the WEGO2.0 website (https://wego.genomics.cn/) [38]. Protein-protein interactions (PPIs) were predicted using the STRING database (https://string-db.org/). The combined score >0.8 in the STRING database was used to confirm the interaction network.

### 4.5. Gene Expression Analysis

To analyze the expression patterns of *TaZF-HD* genes in different tissues and stress conditions, the expression data were obtained from expVIP (http://www.wheat-expression.com/) [39]. The TPM (transcripts per million) values of the *TaZF-HD* genes are presented in Table S5. The heatmap was drawn by TBtools [30].

### 4.6. Plant Materials and Treatments

Wheat seeds of “Chinese Spring” were germinated on moist filter paper at 25/18 °C (day/night) with a photoperiod of 16 h/day. Wheat seedlings were grown in a hydroponic culture for two weeks and root, stem and leaf tissues of the wheat seedlings were collected. For the abiotic stress treatment, seedlings were exposed to 20% PEG 6000 (*w*/*v*), high salinity (300 mM NaCl), high temperature (42 °C) and cold (4 °C) conditions as described previously [40]. In each treatment, the leaf tissues were collected every 12 h for 36 h. All samples were frozen in liquid nitrogen and stored at −80 °C.

### 4.7. RNA Isolation and Real-Time PCR Analysis

An EasyPure Plant RNA Kit (TransGen) was used to isolate total RNA from each frozen sample and the first-strand cDNA was synthesized from total RNA (1 μg) by using EasyScript One-Step gDNA Removal and cDNA Synthesis SuperMix (TransGen) according to the manufacturer’s instructions. The sequence was amplified using gene-specific primers (Table S7) with TransTaq-T DNA Polymerase (TransGen) and the *actin* gene was used as an internal control. The real-time PCR cycling parameters were 94 °C for 30 s followed by 45 cycles at 94 °C for 5 s and 55 °C for 30 s with a melting curve analysis from 60 °C to 90 °C at a rate of 0.5 °C/5 s. All reactions were performed in triplicate to ensure the reproducibility of the results.

## Data Availability

The public RNA-seq data were obtained from expVIP (http://www.wheat-expression.com/).

## Supplementary Materials

The following are available online at https://www.mdpi.com/2223-7747/10/3/593/s1, Figure S1: Conserved domains of TaZF-HD proteins in wheat. Figure S2: Chromosomal localizations of *TaZF-HD* genes in *T. aestivum*. Figure S3: The expression levels of *TaZHD3* and *TaZHD19* under drought, PEG, heat and cold stress. The log_2_ of the TPM values were calculated by RNA-seq data to show the expression levels of the *TaZHD3* and *TaZHD19* genes under drought, PEG, heat and cold stress in wheat. Table S1: The characteristics of *ZF-HD* genes in wheat. Table S2: *ZF-HD* genes used in the phylogenetic tree construction. Table S3. Paralogous *ZH-HD* gene pairs among *T. aestivum*. Table S4: Orthologous relationships between *TaZF-HD* genes in *T. aestivum* with other *ZF-HD* genes in *T. urartu*, *Ae. tauschii*, *B. distachyon* and *O. sativa*. Table S5: The expression levels of *TaZF-HD* genes in different tissues of wheat. Table S6: The protein-protein interaction network between TaZF-HDs and other proteins in wheat. Table S7: Real-time PCR primers of *TaZF-HD* genes.
