# Supplementary figures and images for: Zinc Finger-Homeodomain Transcriptional Factors (ZF-HDs) in Wheat (Triticum aestivum L.): Identification, Evolution, Expression Analysis and Response to Abiotic Stresses
Source: Plants (Basel). 2021 Mar 22;10(3):593. doi: 10.3390/plants10030593 (PMC8004245; doi:10.3390/plants10030593)

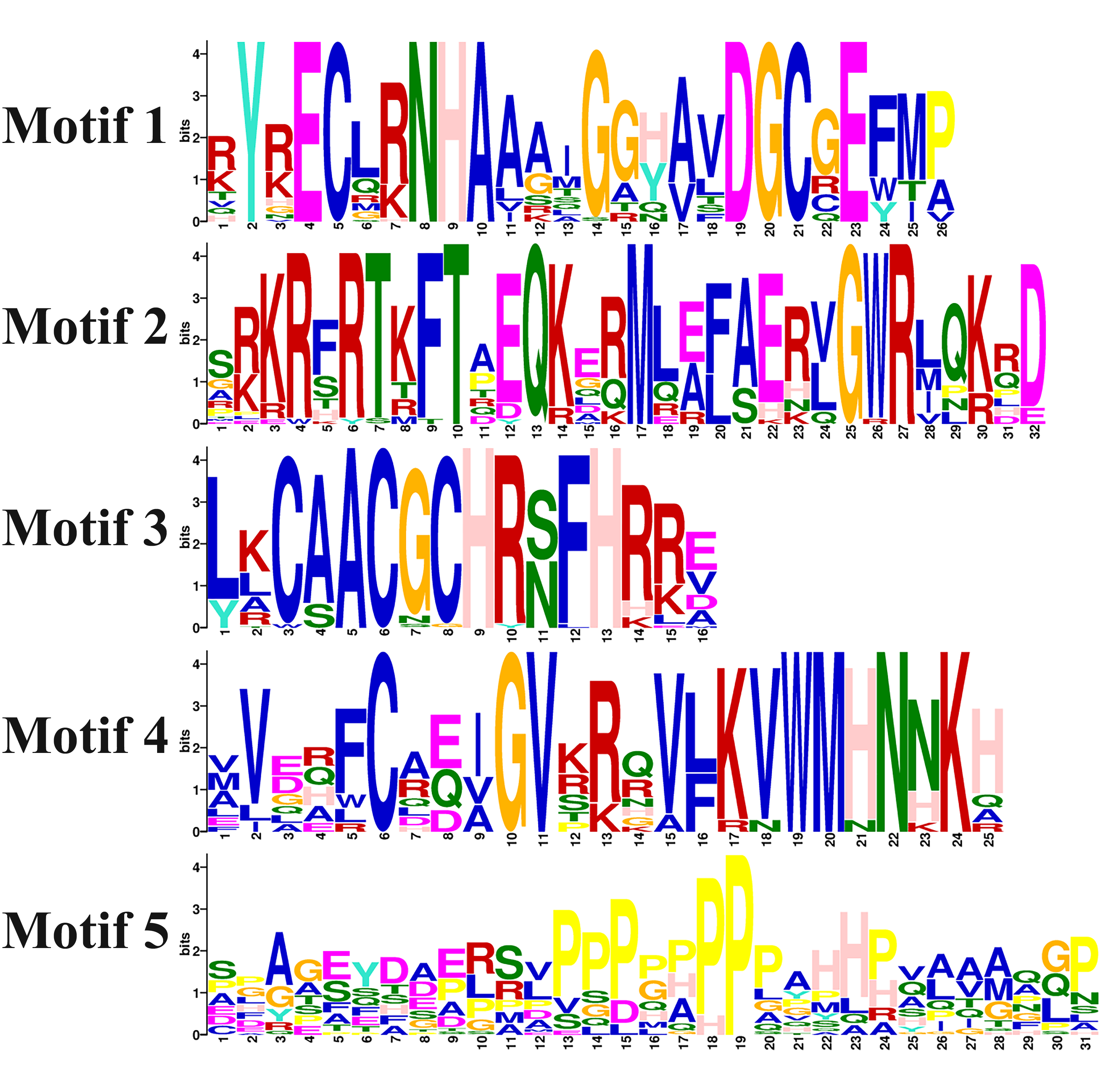

Supplement: Supplementary file 1 [file plants-10-00593-s001.zip › plants-1129384-supplementary/Supplementary File/Fig. S1.tif]

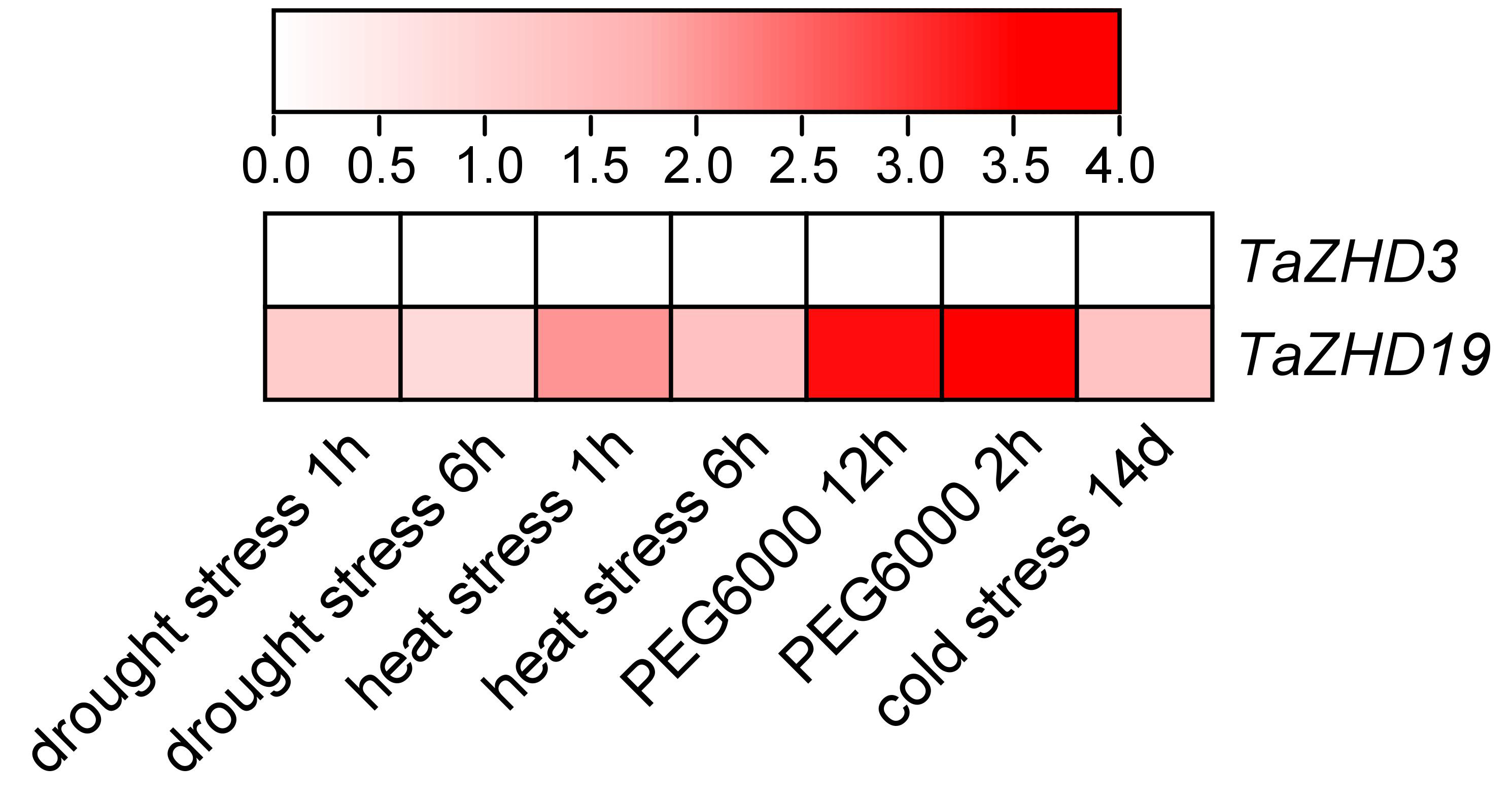

Supplement: Supplementary file 1 [file plants-10-00593-s001.zip › plants-1129384-supplementary/Supplementary File/Fig. S3.jpg]
